# Fascin in Cell Migration: More Than an Actin Bundling Protein

**DOI:** 10.3390/biology9110403

**Published:** 2020-11-17

**Authors:** Maureen C. Lamb, Tina L. Tootle

**Affiliations:** Anatomy and Cell Biology Department, Carver College of Medicine, University of Iowa, Iowa City, IA 52242, USA; maureen-lamb@uiowa.edu

**Keywords:** fascin, migration, cancer metastasis, mechanotransduction, actin, microtubules, LINC Complex, nuclear actin, nucleolus, prostaglandins

## Abstract

**Simple Summary:**

Cell migration is an essential biological process that regulates both development and diseases, such as cancer metastasis. Therefore, understanding the factors that promote cell migration is crucial. One of the factors known to regulate cell migration is the actin-binding protein, Fascin. Fascin is typically thought to promote cell migration through bundling actin to form migratory structures such as filopodia and invadapodia. However, Fascin has many other functions in the cell that may contribute to cell migration. How these novel functions promote cell migration and are regulated is still not well understood. Here, we review the structure of Fascin, the many functions of Fascin and how they may promote cell migration, how Fascin is regulated, and Fascin’s role in diseases such as cancer metastasis.

**Abstract:**

Fascin, an actin-binding protein, regulates many developmental migrations and contributes to cancer metastasis. Specifically, Fascin promotes cell motility, invasion, and adhesion by forming filopodia and invadopodia through its canonical actin bundling function. In addition to bundling actin, Fascin has non-canonical roles in the cell that are thought to promote cell migration. These non-canonical functions include regulating the activity of other actin-binding proteins, binding to and regulating microtubules, mediating mechanotransduction to the nucleus via interaction with the Linker of the Nucleoskeleton and Cytoskeleton (LINC) Complex, and localizing to the nucleus to regulate nuclear actin, the nucleolus, and chromatin modifications. The many functions of Fascin must be coordinately regulated to control cell migration. While much remains to be learned about such mechanisms, Fascin is regulated by post-translational modifications, prostaglandin signaling, protein–protein interactions, and transcriptional means. Here, we review the structure of Fascin, the various functions of Fascin and how they contribute to cell migration, the mechanisms regulating Fascin, and how Fascin contributes to diseases, specifically cancer metastasis.

## 1. Introduction

Fascin is an actin-binding protein that promotes different modes of cell migration, including during embryonic development and cancer metastasis [1,2]. One means by which Fascin promotes migration is by crosslinking or bundling actin filaments together [3,4]. Through this function, Fascin aids in the formation of many actin-rich migratory structures such as filopodia [5,6,7], lamellipodia [5], stress fibers [8], and microspikes [9,10]. Fascin also localizes to actin-rich adhesive structures such as focal adhesions [8], invadopodia [11], and podocytes [12,13]. Due to its roles in cell migration, Fascin has frequently been studied in the context of cancer. Fascin is absent in adult epithelial tissue [14] but is highly expressed in many types of carcinomas. High Fascin expression is correlated with poor prognosis and increased metastasis (reviewed in [4]). For this reason, Fascin is a prognostic biomarker and a potential therapeutic target of metastatic carcinomas [2,4]. While studies on Fascin have centered around Fascin’s canonical actin bundling function, Fascin also has non-canonical, non-bundling functions in the cell. In this review, we discuss Fascin’s structure and expression, explore how both the canonical and non-canonical functions of Fascin promote migration, examine the various mechanisms regulating Fascin, and consider how the different functions of Fascin may contribute to diseases.

## 2. Fascin Structure, Expression, and Functions

Fascin promotes cell migration not only through its conserved actin bundling activity, but also by its non-canonical functions. In this section, we review the structure of Fascin, how it bundles actin, and Fascin’s expression pattern during development and in adult tissues. Then, we discuss how Fascin promotes cell migration through both its canonical actin bundling function and non-canonical functions: modulating the activity of other actin-binding proteins [8,15,16], binding to microtubules [17], interacting with the Linker of the Nucleoskeleton and Cytoskeleton (LINC) Complex [18], and having nuclear roles [19].

### 2.1. Structure of Fascin

**Figure 1:**
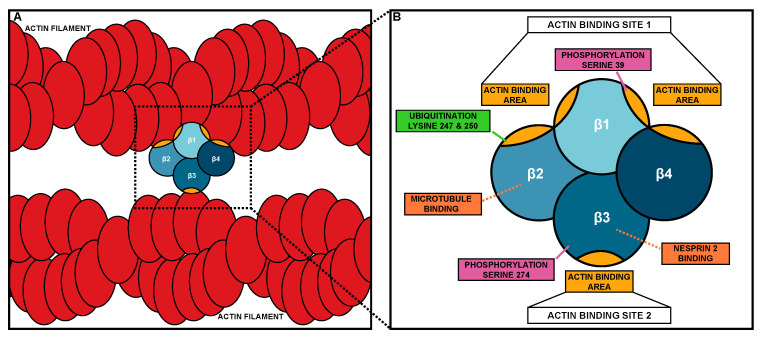
Fascin bundling mechanism and actin-binding domains. (**A**) Schematic of Fascin bundling two actin filaments (red). (**B**) Schematic of the domains and binding site of Fascin. The four β-trefoil domains of Fascin are in different shades of blue. Actin-binding areas of Fascin are in gold. In B, the different protein interaction sites and post-translational modifications are labeled.

Fascin was initially discovered in sea urchins [20] and later found in Drosophila [21,22], Xenopus [23], mouse [24], and humans [25,26]. Fascin is a globular protein of approximately 55kDa and is comprised of four β-trefoil domains (Figure 1) [26]. Each β-trefoil domain contains six two-stranded β-hairpins oriented in a trifold symmetry [27,28]. The four β-trefoils of Fascin are arranged into two lobes (Figure 1B) [26]. This structure allows a monomer of Fascin to bundle actin, while most actin bundlers function as dimers (Figure 1A). Fascin has three distinct surface areas able to bind actin; two larger actin-binding areas are in the clefts of β-trefoils 1 and 2, and β-trefoils 1 and 4, while a third smaller area is in β-trefoil 3 (Figure 1A,B) [29,30]. Based on cryoEM analysis of actin bundled by Fascin in filopodia, the two larger actin-binding areas (referred to as actin-binding site 1) bind to a single actin filament, while the actin-binding area in β-trefoil 3 (referred to as actin-binding site 2) binds to a second actin filament. The two sites of actin bundling are approximately 5 nm apart, which along with Fascin being monomeric, causes Fascin to be the tightest actin bundler with approximately 8.1 nm between actin filaments [29,31]. Recent X-ray crystallography, along with the systematic mutagenesis of Fascin, reveals that mutation in one of the actin-binding sites impairs the other site’s actin-binding activity, suggesting there is coordinated regulation between Fascin’s actin-binding sites [29]. 

### 2.2. Expression and Bundling Function of Fascin

Humans and other vertebrates have three Fascin isoforms (Fascin-1, Fascin-2, and Fascin-3) that each have distinct expression patterns and functions. Fascin-1 (FSCN1) is the most widely studied and is expressed in mesenchymal and nervous tissues during development [1]. Fascin-2 has 56% homology to Fascin-1 and is expressed in the inner and outer segments of photoreceptors and in the stereocilia of the hair cells [32,33,34], where it bundles actin to stabilize the protrusive structures these cells form [32,33]. Moreover, mutations in Fascin-2 are associated with retinitis pigmentosa and hearing loss [33,35,36]. Fascin-3 has 29% homology to Fascin-1 and is expressed in the testis and developing spermatozoa, where it plays a role in the terminal elongation of the spermatid head [37]. Interestingly, the area surrounding actin-binding site 1 is highly conserved across all three Fascins [37]. Thus, the different isoforms of Fascin bundle actin in different tissues and structures.

During mouse embryonic development, Fascin-1 (subsequently referred to as Fascin) is widely expressed [1,14,38]. Indeed, Fascin is expressed in the central and peripheral nervous system, the neuroepithelium, developing somites, and the mesenchyme of limb buds [1]. While Fascin is expressed in a wide variety of tissues, it is largely dispensable for mouse development. Fascin knockout mice are not embryonic lethal but have increased neonatal lethality [38]. These mice display defects in the central nervous system, including a notable enlargement of the lateral ventricles [38]. The neonatal lethality is attributed to poor feeding and breathing after birth, but the consequences of the other abnormalities are not well understood [38]. Loss of Fascin in these mice is potentially compensated for by the other two Fascins (Fascin-2 and Fascin-3); however, a triple knockout mouse model has not been created. While Fascin is largely dispensable for mouse embryonic development, its expression patterns are largely conserved in human tissues.

In humans, Fascin is more widely expressed during development than in the adult. While Fascin is largely absent from adult epithelial tissues, Fascin is expressed in neurons, the glomerulus of the kidney, the adrenal gland, the basal layer of the skin, and immune cells of adults [14]. In particular, Fascin is highly expressed during dendritic cell maturation [39]. Dendritic cells are highly motile cells and play an important role in innate and adaptive immunity [39]. Loss of Fascin impairs dendritic cell maturation and therefore motility [39]. During embryonic and fetal development, Fascin is widely expressed in the nervous system, including neuroblasts, neural crest cells, melanoblasts, mesenchymal tissues, microcapillary endothelial cells, gastrointestinal tract, and antigen-presenting dendritic cells [14,40,41,42,43,44]. A common feature of many of these cell types is that they undergo significant cell migrations, highlighting the importance of Fascin in promoting cell migration. For example, Fascin-expressing neuroblasts of the subventricular zone in the postnatal brain migrate along the rostral migratory stream to the olfactory bulb [44]. 

The role of Fascin in migration is conserved across organisms. Indeed, even in lower eukaryotes, Fascin-expressing cells undergo significant migrations. For example, in sea urchins, Fascin is expressed in coelomocytes [45]. Coelomocytes are migratory phagocytic leukocytes that perform essential immune functions [45]. In these cells, Fascin bundles actin to promote filopodia formation and migration [45]. In zebrafish, Fascin promotes migration by forming filopodia in a subset of neural crest cells [40]. In Drosophila, the macrophage-like hemocytes that migrate throughout the developing embryo highly express Fascin, which promotes their migration through filopodia formation [46]. Fascin is also highly expressed in the migratory border cells of the Drosophila ovary [22] and is essential for their on-time collective migration [47]. Specifically, Fascin promotes both delamination of the border cell cluster from the epithelium and the proper formation of protrusions [47]. These examples highlight the conservation of Fascin in promoting cell migration across organisms. 

### 2.3. Non-Canonical Roles of Fascin

**Figure 2:**
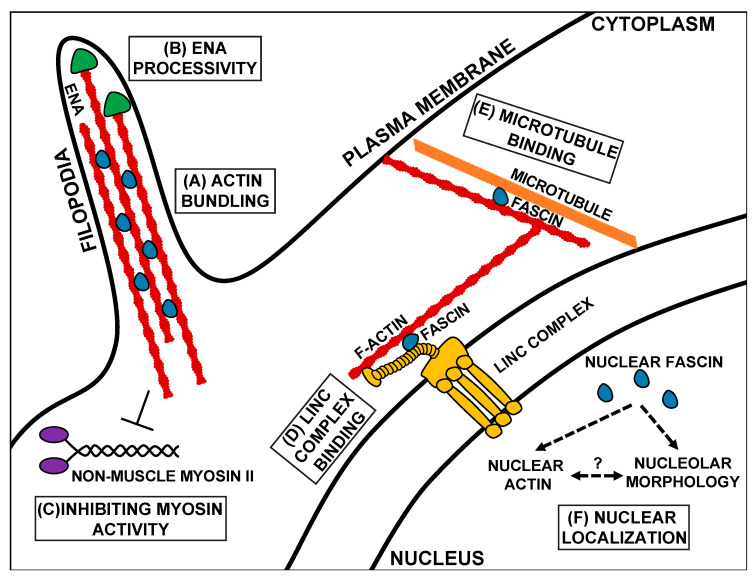
Schematic of the canonical and non-canonical functions of Fascin. Canonically, Fascin (blue) bundles actin filaments (red) to form structures such as filopodia (**A**). Fascin also regulates other actin-binding proteins, such as increasing the processivity of the actin elongating factor, Ena (green, **B**), and inhibiting Non-muscle Myosin II (purple) activity (**C**). At the nuclear periphery, Fascin mediates mechanotransduction by interacting with the Linker of the Nucleoskeleton and Cytoskeleton (LINC) complex (gold, **D**). In addition to binding actin, Fascin also binds to microtubules (orange, **E**). Fascin also localizes to the nucleus, where it likely regulates nuclear actin and nucleolar morphology (**F**). Schematic is not drawn to scale.

While Fascin’s role in promoting migration, especially in the context of cancer metastasis, is primarily attributed to its function as an actin bundler, research has uncovered many other functions of Fascin. These new functions not only demonstrate that Fascin promotes cell migration through multiple mechanisms and pathways (Figure 2) but are new potential therapeutic targets to prevent cancer metastasis. 

In addition to forming cellular protrusions and extensions, the actin bundling function of Fascin regulates other actin-binding proteins, including Enabled (Ena) and Non-muscle Myosin II (Figure 2B,C). Ena is an actin elongation factor that binds to the plus end of actin filaments and promotes polymerization [48]. When actin filaments are bundled in vitro by either human or Drosophila Fascin (Singed), Ena has increased processivity, meaning it stays associated with and polymerizes actin filaments for longer periods of time (Figure 2B) [15,16]. This increased processivity is not observed with other actin bundlers [15,16]. Similarly, during Drosophila border cell migration, Fascin and Ena genetically interact to promote on-time migration [47]. This interaction suggests that Fascin increases Ena processivity in the border cells to mediate on-time migration, potentially by promoting protrusion formation in the cluster [47]. In addition to regulating Ena, Fascin also regulates Non-muscle Myosin II activity in vitro [8] (subsequently referred to as Myosin II). Specifically, Fascin limits Myosin II’s ATP consumption and decreases Myosin II’s motor activity (Figure 2C) [8]. This regulation may occur by Fascin bundling actin filaments and precluding Myosin II’s ability to bind to the filaments [8]. Since Myosin II can alter both cell contractility and cell stiffness, it is an essential component in both migrating cells and their substrate [49]. Thus, Fascin’s actin bundling activity promotes migration by not only by mediating the formation of cell migratory structures but also by modulating the activity of actin-binding proteins. 

Fascin’s bundling activity also controls actin filaments outside of cell migratory structures. For example, Fascin mediates the remodeling of mitochondrial actin filaments to control mitochondrial oxidative phosphorylation. In lung adenocarcinomas, Fascin stabilizes filamentous actin around mitochondria, which regulates mitochondrial fission and fusion as well as promotes metastasis [50]. 

Another means by which Fascin contributes to migration is by binding to and regulating microtubules [17]. Microtubules directly interact with Fascin via β-trefoil 2, which likely blocks Fascin’s actin-binding site 1 (Figure 1B or Figure 2E) [17]. Fascin’s interaction with microtubules promotes cell migration by regulating microtubule dynamics [17]. Blocking the microtubule–Fascin interaction impairs in vitro cell migration by increasing the size and reducing the turnover of focal adhesions [17]. Fascin promotes microtubule-dependent focal adhesion disassembly, and therefore cell migration, by activating the FAK-Src pathway [17]. These data reveal that Fascin interacts with microtubules to regulate focal adhesions to promote cell migration.

Fascin also localizes to the outside of the nucleus in Drosophila nurse cells and mammalian cells [19]. This perinuclear localization is reminiscent of the LINC Complex, which is a key mediator of mechanotransduction [51,52]. The LINC Complex is composed of KASH (Klarischt, ANC-1, Syne Homology) and SUN (Sad1p, UNC-84) proteins. KASH proteins interact with cytoskeletal filaments in the cytoplasm and extend through the outer nuclear envelope to connect to SUN proteins. SUN proteins extend from the inner nuclear envelope into the nucleus, where they bind to the nuclear lamina [51,52]. This structure allows the transmission force from the outside of the cell, through the cytoskeleton, to the nucleus and regulates the nuclear shape and position during cell migration [51,52,53,54]. Fascin binds directly to the KASH protein, Nesprin 2, through β-trefoil 3 (Figure 1B or Figure 2D). This interaction allows Fascin to bind to the LINC Complex and to actin through its actin-binding site 1, thus connecting the LINC Complex to the actin cytoskeleton [18]. While Nesprins can directly bind to actin filaments, interaction with Fascin may aid in strengthening this connection, particularly for invasive migrations [18]. Disruption in this interaction leads to significant impairments in nuclear deformation during single-cell invasive migrations [18]. This work suggests that in addition to bundling actin, Fascin plays a key role in regulating mechanotransduction, which is critical for cell migration. 

Fascin may also contribute to cell migration independent of its interaction with cytoskeletal filaments. Indeed, while Fascin primarily localizes in the cytoplasm, it also localizes to the nucleus (Figure 2F) [19]. This nuclear localization is likely conserved across organisms, as it is observed in both Drosophila nurse cells and mammalian cell lines [19]. While the functions of nuclear Fascin remain poorly understood, it may regulate nuclear actin. Indeed, complete loss of Fascin reduces, while an overexpression of Fascin increases the frequency of Drosophila nurse cells with high levels of one form of nuclear actin [55]. Changes in nuclear actin regulation can alter gene expression (reviewed in [56]), which can impinge upon processes such as cell migration. Thus, it is tempting to speculate that nuclear Fascin could promote cell migration by regulating nuclear actin and, thereby, transcription factor activity and gene expression. For example, nuclear actin regulates transcription by modulating several transcription factors, such as myocardin-related transcription factor A (MRTF, also referred to as megakaryocytic acute leukemia (MAL)). Specifically, nuclear actin regulates MRTF’s nuclear localization and activity [57]. MRTF promotes many types of cell migration [58]. Another potential role of nuclear Fascin is to regulate the functions of the nucleolus [19]. The nucleolus forms by phase separation, meaning that its function is tightly associated with its structure. Indeed, disruption of its functions results in severe alteration in its morphology and vice versa [59]. Fascin promotes proper nucleolar morphology in both Drosophila nurse cells and mammalian cell lines [19]. In addition to producing ribosomes, the nucleolus is an essential metabolic regulator and responder to cellular stress, both of which can impinge upon cell migration. Interestingly, nuclear actin localizes to the nucleolus and regulates nucleolar functions [60]; these data suggest that Fascin may regulate nuclear actin to influence the nucleolar structure and function. Supporting a nucleolar role for Fascin, a proteomic analysis of Fascin-interacting proteins in laryngeal squamous cell carcinomas identified several nucleolar interacting partners involved in ribosomal RNA processing [61]. Lastly, nuclear Fascin interacts with a histone methyltransferase subunit, RbBP5, to promote histone3 Lys4 trimethylation at target gene promoter regions [62]. Thus, one nuclear function of Fascin is to regulate histone modifications and transcription [62]. Together, these findings highlight how Fascin has diverse and poorly understood nuclear functions and that further studies are needed to determine how nuclear Fascin functions during cell migration. 

Overall, Fascin is a highly conserved regulator of cell migration. Fascin is the tightest actin bundler in the cell and mediates the formation of cell migratory structures, such as filopodia. In addition to bundling actin, Fascin has many non-canonical functions, including modulating the activity of actin-binding proteins [8,15,16], regulating microtubule dynamics [17], mediating mechanotransduction via the LINC Complex [18], and acting within the nucleus [19]. Therefore, Fascin influences cell migration through its canonical actin bundling activity, but it likely also promotes cell migration through one or more of these other functions.

## 3. Regulation of Fascin

Since Fascin has multiple functions, tight regulation of these functions is necessary to ensure cell migration. Fascin is regulated by post-translational modifications, upstream pathways, protein–protein interactions, and transcriptional means. However, very little is known about how these different regulatory mechanisms coordinate the different functions of Fascin. This section discusses the mechanisms regulating Fascin and how they influence both Fascin’s actin bundling and non-canonical functions.

### 3.1. Post-Translational Modifications

The most well-known mechanism of regulating Fascin is through phosphorylation. Fascin phosphorylation has been investigated both in human cell lines and Drosophila. Fascin has two known phosphorylation sites. One site is serine 39 (S39, Drosophila S52), which is in actin-binding site 1 on β-trefoil 1 (Figure 1B). Thus, it is not surprising that phosphorylation at S39 inhibits Fascin’s actin bundling function [63,64]. Protein Kinase C (PKC) phosphorylates Fascin at S39, and this phosphorylation promotes Fascin binding to PKC; this is observed both in vitro and in vivo [65]. The other phosphorylation site was first identified in Drosophila (S289) but is conserved in humans (S274) [66]. This site is on β-trefoil 3 near actin-binding site 2 (Figure 1B). Both the phospho-mutant and phospho-mimetic of S289 in Drosophila impair Fascin’s actin bundling activity in vitro and alter Fascin’s localization along actin bundles in Drosophila hemocytes and mammalian cell lines. However, the phospho-mutant can rescue filopodia formation and migration in Drosophila hemocytes and actin bundle formation in Drosophila nurse cells [66]. These data suggest that Fascin requires dynamic cycles of phosphorylation and dephosphorylation to properly form filopodia and promote migration. It remains unknown what kinase mediates phosphorylation at S274 (Drosophila S289) and what phosphatases regulate both phosphorylation sites; however, calcineurin is able to dephosphorylate Fascin in vitro [64]. While the phosphorylation of Fascin has largely been investigated in the context of Fascin’s actin bundling function, phosphorylation also regulates Fascin’s non-canonical roles. 

The phosphorylation of Fascin controls its microtubule binding, nuclear localization, and LINC Complex interaction. Specifically, the phosphorylation of S274 promotes Fascin’s interaction with microtubules in cultured cells [17]. In Drosophila, phosphorylation at S289 may promote, while dephosphorylation at S52 (mammalian S39) may inhibit Fascin’s nuclear localization (Groen and Tootle, unpublished observations). Finally, the phosphorylation of S39 (Drosophila S52) promotes Fascin’s perinuclear localization and binding to Nesprin-2, the cytoplasmic portion of the LINC Complex, in both mammalian cells and Drosophila nurse cells [18]. Interestingly, binding to Nesprin 2 disrupts Fascin’s actin-binding site 2 and phosphorylation at S39 impairs actin-binding site 1 (Figure 1B); therefore, how Fascin connects the LINC Complex to the actin cytoskeleton is unclear. Potentially, Fascin is able to bind to an actin filament using part of actin-binding site 1, such as the actin-binding area in the cleft between β-trefoil 1 and 2, while still interacting with the LINC Complex via β-trefoil 3. Together, these data suggest that phosphorylation coordinately regulates multiple functions of Fascin.

In addition to phosphorylation, Fascin has other post-translational modifications. In vitro, Fascin is monoubiquitinated at two different lysine residues, lysine 247 (K247) and lysine 250 (K250). These residues reside in Fascin’s actin-binding site 1 on β-trefoil 2 (Figure 1B) [67]. Ubiquitination at these sites impairs bundle formation and accelerates bundle disassembly [67]. In vitro, this ubiquitination is mediated by the E3 ligase, Smurf1 [67]. However, how ubiquitination regulates both the canonical and non-canonical functions of Fascin is not fully understood. 

### 3.2. Regulation by Prostaglandin Signaling

An upstream regulator of Fascin is prostaglandin (PG) signaling. PGs are short-range lipid signaling molecules that regulate various physiological processes through numerous downstream targets [68,69]. One group of downstream targets of PG signaling is the actin cytoskeleton [70,71,72,73,74,75,76]. Studies using Drosophila oogenesis uncovered that PG signaling regulates numerous actin-binding proteins, including Fascin, Ena, and Myosin II [71,77,78]. In Drosophila, PG signaling positively regulates Fascin to mediate actin bundle formation and cortical actin integrity in the nurse cells [71] and promotes the collective migration of the border cells [79]. In these contexts, PG signaling likely regulates both the actin-bundling activity of Fascin, and its roles in regulating Ena and Myosin II. Indeed, PGs act upstream of Ena to promote actin filament formation [77,78] and control Myosin II-dependent cellular contraction [78]. During Drosophila oogenesis, PG signaling is required for Fascin’s perinuclear localization [19]. As this perinuclear localization also requires a functional LINC Complex [18], PG signaling likely regulates Fascin’s interaction with the LINC Complex. PG signaling also regulates the nuclear localization of Fascin [19]. Specifically, PG signaling temporally regulates the levels of nuclear Fascin, and through Fascin, it regulates nucleolar morphology [19]. These data support the model that PG signaling coordinately regulates the multiple functions of Fascin to mediate developmental processes. While the mechanisms by which PG signaling regulates Fascin remain unknown, PGs do not regulate Fascin expression [71,80]. Furthermore, unpublished observations suggest that PGs modulate Fascin’s post-translational modifications. Notably, the kinase known to phosphorylate Fascin, PKC, and the putative phosphatase, calcineurin, are both downstream targets of PGs [68,81]. Further studies are needed to fully elucidate how PG signaling regulates the different functions of Fascin. This regulation of Fascin is likely important in other contexts, such as cancer metastasis.

### 3.3. Protein–Protein Interactions

Protein–protein interactions are another means of regulating Fascin. As mentioned above, PKC phosphorylates and binds to Fascin, which inhibits Fascin’s actin bundling function and mediates the localization of the complex to cell margins [65]. Intriguingly, the Fascin–PKC interaction contributes to myoblast migration on fibronectin [65], and both Fascin’s actin bundling and PKC interaction promote migration in human colon carcinoma cells [6]. The Fascin–PKC interaction is positively regulated upstream by the Rho GTPase, Rac, through its effector Pak1 in human colon carcinoma cells [82]. Thus, PKC not only regulates Fascin by phosphorylation but also by directly binding to Fascin.

Filopodia formation and cell migration are also mediated by Fascin’s direct interaction with other partners. For example, Fascin binds to p-Lin-11/Isl-1/Mec-3 kinases (LIMK) to promote filopodia formation downstream of Rho [83]. Fascin also promotes filopodia formation in vitro through its interaction with Daam1, an actin polymerization protein [84]. Additionally, by directly binding to the p75 neurotrophin receptor, Fascin promotes migration of melanoma cells [85]. This interaction is inhibited by the phosphorylation of S39 on Fascin [85]. Fascin also interacts with the vesicle trafficking protein, Rab35 [86]. Rab35 may recruit Fascin to actin bundling sites, as disruption of the interaction impairs filopodia formation in cultured cells [86]. These data indicate numerous proteins bind to and regulate Fascin and suggest that these means of regulation may occur in a cell context-specific manner. 

### 3.4. Transcriptional Regulation

In many instances, Fascin expression is upregulated to induce or promote the migration of cells. For example, Fascin expression is upregulated during dendritic cell maturation through a core promoter region containing a cyclic adenosine monophosphate (cAMP) response element, an enhancer, and a distal repressor [87,88]. These elements regulate Fascin expression, which promotes dendritic cell migration and T-cell activation [89]. In Drosophila, Fascin expression is also transcriptionally upregulated in the migrating border cells [22,90]. The border cells are specified through Janus kinase (JAK)-signal transducer and activator of transcription (STAT) signaling [91]. JAK/STAT signaling induces the expression of the CCAAT-enhancer-binding proteins (C/EBP) transcription factor, Slbo, which then promotes the expression of hundreds of migratory genes in the border cells [90,91]. One of Slbo’s targets is Fascin [90]. Fascin is dramatically upregulated in the border cells during their specification, remains highly expressed throughout the migration, and is critical for on-time migration [22,47,90]. These studies demonstrate that transcriptional expression is one of the mechanisms by which Fascin is regulated to control cellular migrations. 

The transcriptional regulation of Fascin has also been extensively studied in the context of cancer. Understanding how Fascin, which is not typically expressed in adult epithelial tissues, becomes highly expressed in malignant carcinomas is of particular interest [3,4]. A study of Fascin’s promoter identified region −219/+114 as having strong transcriptional activity [92]. The cAMP response element-binding protein (CREB) and aryl hydrocarbon receptor binding motifs in this region are potent regulators of *fascin* transcription in colon, breast, and lung carcinomas [92,93,94]. Specifically, mutations in the binding sites significantly decreased Fascin expression in cancer cell lines [92]. There is also evidence that Fascin is a putative target of β-catenin–T-cell factor (TCF) signaling in colon and gastric carcinomas [95,96], Transforming growth factor beta (TGFβ) signaling in breast carcinomas [97,98] and STAT3/ Nuclear factor kappa B (NF-kB) signaling in breast and gastric carcinomas [99,100]. The specific signaling mechanisms that induce high Fascin expression in malignant carcinomas are likely dependent on the cellular context. However, understanding the mechanisms that increase Fascin expression in different cancers is crucial for understanding Fascin’s roles in promoting cancer progression. 

Another mechanism controlling the expression of Fascin is microRNAs (miRs). miRs are small, non-coding RNA molecules that post-transcriptionally regulate gene expression. Two microRNAs, miR133 and miR145, inhibit Fascin expression, and they are downregulated in many types of cancer, including breast, bladder, esophageal squamous cell, prostate, and colorectal [101,102,103,104,105,106,107,108,109,110]. In these cancers, the downregulation of these microRNAs leads to increased Fascin expression, which is linked to increased cell migration and invasion [101,102,103,104,105,106,107,108,109,110]. Thus, post-transcriptional regulation is a critical mechanism of controlling the expression of Fascin.

These studies indicate that the functions and expression of Fascin are regulated through various mechanisms, including post-translation modifications, upstream regulation, and expression. Given its canonical and non-canonical functions, Fascin likely requires many layers of regulation to ensure its proper function. Further investigation into how Fascin’s many functions are regulated and coordinated in a cell is warranted.

## 4. Fascin and Disease

Given Fascin’s critical roles in cell migration, it contributes to many diseases and pathologies. Here, we discuss the well-studied roles of Fascin in cancer and its roles in other diseases including wound healing and neurological disorders; we also highlight how the non-canonical functions of Fascin may contribute to these diseases. 

### 4.1. Fascin and Cancer

Fascin’s role in cancer has been extensively studied. Whereas most adult epithelial tissues do not express Fascin [1], Fascin expression is highly upregulated in many types of carcinomas including breast, colon, gastric, and oral squamous cell carcinomas [2]. In these cancers, increased Fascin expression is linked to increased aggressiveness and high mortality [2,4,111]. Additionally, high Fascin expression is associated with specific subtypes of highly aggressive forms of cancers, such as serrated colorectal adenocarcinoma and triple-negative/basal breast carcinomas [112,113,114,115,116]. Thus, Fascin is a common biomarker for aggressive carcinomas, can be used as a histological marker of particular cancer subtypes, and may be a useful therapeutic target, given its low expression in non-malignant adult epithelial tissues [2]. In fact, Phase I clinical trials have begun testing a Fascin inhibitor (NP-G2-044) to treat metastatic carcinomas [117]. While Fascin is not expressed in adult epithelial tissue, it is expressed in other adult tissues [14], raising the concern that Fascin inhibitors may have negative side effects. Specifically, the inhibition of Fascin may cause neuronal, kidney, endocrine, wound healing, and immune defects. 

The main mechanism by which Fascin is thought to drive cancer progression is by promoting cancer migration, invasion, and metastasis [2,4]. In vitro, Fascin increases cancer cell migration in oral squamous carcinoma cells [53] and promotes filopodia formation in colon carcinoma cells [6]. In breast cancer cells, Fascin regulates invadopodia formation and stability to promote invasion in a 3D environment [10]. Fascin also promotes invasion in other model systems; for example, Fascin increases invasion of colorectal carcinoma cells in zebrafish [118]. This suggests a conserved function of Fascin in cancer. Furthermore, Fascin promotes cancer metastasis. In an in vivo mouse model of breast cancer, increased Fascin expression promotes metastasis to the lung [119]. Increased Fascin expression also promotes metastatic colonization in pancreatic [120] and colorectal carcinomas [117]. Further, Fascin promotes metastatic expansion, in part by controlling metabolism and contributing to a de-differentiated and more stem-like state [121]. Additionally, Fascin has been found in mesothelioma cell-derived exosomes [122]. Exosomes are small vesicles secreted by the tumor, primary or metastatic, to help evade immune-directed destruction. Further, Fascin is a critical mediator of the metastatic process of self-seeding. Self-seeding is when circulating tumor cells recolonize their primary tumor [123]. Fascin knockdown in circulating tumor cells impairs the self-seeding process [123]. Together, these and numerous other studies demonstrate a clear role of Fascin in promoting cancer cell migration, invasion, and metastasis. 

Interestingly, Fascin also regulates cancer initiation and proliferation. Indeed, inducing Fascin expression in colon cells significantly increased tumor initiation in a mouse model [124]. Furthermore, these tumors progress more rapidly and lead to the formation of invasive adenocarcinomas [124]. Fascin also regulates cancer cell proliferation in breast cancer [125] and melanoma cells [41]. How Fascin regulates processes such as cancer initiation and proliferation is still unclear. 

Fascin’s role in cancer is well documented and studied; however, research has focused on Fascin’s actin bundling function as the main mechanism contributing to cancer progression. As described above, Fascin has several non-canonical functions in the cell, and it is likely that these functions contribute to cancer migration and metastasis. One such non-canonical function of Fascin is to bind to and regulate microtubules. Changes in microtubule stability and expression occur in various cancers and can affect cancer metastasis [126]. For example, increased microtubule polymerization promotes breast cancer cell invasion in response to hypoxia [127]. As Fascin regulates microtubule dynamics in cultured cells [17], Fascin may act similarly in cancer cells. Indeed, a study found that in breast cancer cells, overexpression of Fascin promotes metastasis independent of its actin-bundling activity, and these cells exhibit altered microtubule dynamics [128]. These data suggest that Fascin functions in non-canonical ways to regulate cancer metastasis.

Another non-canonical function of Fascin is to mediate mechanotransduction through its interaction with the LINC Complex. The LINC Complex is required for invasive cell migrations, such as when cancer cells invade between neighboring cells and basement membranes during metastasis [51,54]. As disrupting the interaction between Fascin and the LINC Complex impairs single-cell invasive migration [18], Fascin may act within the LINC Complex to regulate mechanotransduction and promote invasive cancer migrations. 

Fascin may contribute to cancer progression by functioning within the nucleus to regulate the nucleolus [19,61], nuclear actin [55], and chromatin accessibility [62]. Fascin regulates nucleolar size and morphology [19]. Changes in nucleolar morphology are a hallmark of cancer cells as nucleoli enlarge to increase ribosome production and, therefore, protein production for increased cell growth, division, and metabolism [129,130]. Many chemotherapies target the nucleolus directly [129]. In addition to ribosome biogenesis, the nucleolus has many other roles such as regulating cell proliferation, differentiation, senescence, and apoptosis, all of which can affect cancer development and progression [129,131,132]. Indeed, in laryngeal squamous cell carcinomas, Fascin interacts with several nucleolar interacting partners involved in rRNA processing [61]. As for Fascin’s regulation of nuclear actin, increased levels of nuclear actin are associated with a malignant phenotype [133]. In breast epithelial cells, increased levels of nuclear actin allow escape from senescence and increase cellular growth [133,134]. Additionally, nuclear actin has various functions, including chromatin regulation, interacting with RNA polymerases, and modulating DNA repair [56]. Dysregulation of any of these functions can contribute to cancer development and progression. Interestingly, in breast cancer cells lines, Fascin directly regulates active histone modifications on genes essential for amino acid metabolism [62]. It will be important to determine if this role of Fascin involves the modulation of nuclear actin, given Fascin’s role in regulating nuclear actin, and nuclear actin’s roles in chromatin modification. Thus, Fascin’s role in localizing to the nucleus and regulating the nucleolus and nuclear actin may play important roles in the pathology of cancer. 

Lastly, Fascin may promote cancer metastasis by regulating mitochondrial oxidative phosphorylation and metabolic stress resistance [50]. In lung adenocarcinomas, Fascin remodels actin filaments surrounding the mitochondria during metabolic stress [50]. This regulates mitochondrial DNA homeostasis, which leads to increased Complex I biogenesis, allowing for increased oxidative phosphorylation [50]. This regulation of oxidative phosphorylation by Fascin allows for metabolic stress resistance in lung adenocarcinoma cells, which enables them to metastasize [50]. Thus, Fascin’s role in regulating metabolic stress resistance may play a crucial role in the ability of cancer cells to metastasize. 

### 4.2. Fascin and Other Diseases

Fascin also plays a role in other conditions besides cancer, including wound healing and neurological diseases. During wound healing, Fascin expression dramatically increases at the wound edge of mouse embryos [135]. In these cells, the SRY-Box Transcription Factors 4 and 11 (SOX4/11) increase Fascin expression, which allows for cell motility and migration during wound repair [135]. In addition to wound healing, multiple proteomic studies implicate Fascin in several neurological diseases. In *stargazer* mutant mice, which are prone to seizures, Fascin expression is reduced [136]. Furthermore, in brain tissue from Alzheimer’s disease patients, there was a two-fold reduction in Fascin expression [137]. While the role of Fascin in neurological diseases is still undetermined, during development, Fascin promotes growth cone formation and extension [138,139] and regulates neuroblast migration in the subventricular zone [44]. Therefore, Fascin likely promotes proper neuron formation and migration, the disruption of which may lead to neurological defects. Together, these data implicate Fascin in regulating diverse disease processes. Further investigation into how Fascin functions in these diseases is warranted. 

Together, these studies support the model that the functions and expression of Fascin must be tightly regulated for normal cellular functions and that dysregulation contributes to diseases and processes such as cancer, wound healing, and neurological disorders. In these contexts, it is likely that Fascin contributes through both its canonical actin bundling function and non-canonical functions. Further investigation into how these other functions of Fascin promote processes, such as cancer metastasis, may provide new therapeutic targets.

## 5. Conclusions

While Fascin is a well-studied regulator of many cell migrations, much remains to be learned about how Fascin functions to promote different types of cell migrations. Fascin is the tightest actin bundler and crosslinks actin filaments in many actin-rich cell migratory structures. In addition to its canonical, actin bundling function, Fascin likely promotes cell migrations through its many non-canonical functions, including regulating actin-binding proteins and mitochondrial function, binding to microtubules, interaction with the LINC Complex, and nuclear localization. Structural analysis determined that Fascin bundles actin in filopodia using three actin-binding areas; however, how Fascin interacts with actin during its non-canonical functions, such as interaction with the LINC Complex or binding microtubules, still remains to be determined. Additionally, while there are many different mechanisms known to regulate Fascin’s canonical actin bundling and non-canonical functions, how the different functions of Fascin are coordinately regulated to promote cell migration remains to be understood. Overall, given the numerous roles of Fascin in development and disease, including cancer metastasis, it will be important to determine how the different functions of Fascin play a role in these contexts.
